# Reduced burden of antibiotic prescription in an italian pediatric primary care clinic during the first wave of COVID-19 pandemic: a shot in the arm for antimicrobial resistance?

**DOI:** 10.1186/s13052-023-01444-5

**Published:** 2023-03-29

**Authors:** Alice Monzani, Giulia Minelli, Ivana Rabbone

**Affiliations:** Division of Paediatrics, Department of Health Sciences, University of Piemonte Orientale, via Solaroli 17, Novara, 28100 Italy

**Keywords:** Antimicrobial resistance, Antibiotic prescription, Children, Pediatrics, COVID-19

## Abstract

Rates of antibiotic-resistant bacteria have increased worldwide over recent years, but the Italian Institute of Health reported a disruption to this trend in 2021 compared with 2020. Children are often recipients of unnecessary antibiotic prescriptions, especially for respiratory tract infections (RTIs). During the initial phase of the COVID-19 pandemic, common RTIs substantially decreased, so it is conceivable that antibiotic prescriptions also reduced during this time. To test this hypothesis, we retrospectively collected data on all visits to a pediatric primary care clinic in Northern Italy from February 20, 2020 to June 2, 2020 and compared data with the same period in 2019. We evaluated the antibiotic prescription rate according to the diagnosis at discharge. While the total number of visits significantly decreased (1335 in 2020 vs. 4899 in 2019), there was only a slight reduction in the antibiotic prescription rate (1039 in 2019, 21.2%, vs. 272 in 2020, 20.4%). However, this corresponded to a 73.8% decrease in the total number of antibiotic prescriptions, with antibiotics for RTI accounting for 69% of the total reduction. It is possible that, at the larger scale, reduced antibiotic prescription in pediatrics during the COVID-19 pandemic resulted in a slight reduction in antimicrobial resistance.

## Main text

There are growing concerns about the threat from antibiotic-resistant bacteria. The Centers for Disease Control and Prevention (CDC) estimates that there was a 15% increase in drug-resistant nosocomial infection rates in 2020, the first year of the COVID-19 pandemic, compared with the previous year, especially with regard to rates of carbapenem-resistant Enterobacterales and carbapenem-resistant *Acinetobacter* [1]. In partial contrast to these data, the Italian Institute of Health (ISS, Istituto Superiore di Sanità) [2] recently reported an overall reduction in antimicrobial resistance rates for eight monitored pathogens (*Staphylococcus aureus, Streptococcus pneumoniae, Enterococcus faecalis, Enterococcus faecium, Escherichia coli, Klebsiella pneumoniae, Pseudomonas aeruginosa*, and *Acinetobacter* spp).

Children are often recipients of unnecessary antibiotics, especially for respiratory tract infections (RTIs), in most cases because these are caused by self-limiting viral conditions. Antibiotics are inappropriately prescribed for RTIs in children due to a lack of readily available tests to assess the viral origin of most RTIs coupled with pediatricians and family doctors seeking to limit parental anxiety about their children. During the first phase of the COVID-19 pandemic, when health measures for SARS-CoV-2 containment were stricter, there was a significant decrease in common RTIs and almost completely suppressed circulation of respiratory viruses other than SARS-CoV-2. It is conceivable that antibiotic prescriptions were similarly reduced. This trend, described in pediatric primary care settings in many countries [3–6], was also confirmed in our pediatric primary care clinic in Northern Italy. We retrospectively collected data on all visits from February 20, 2020 to June 2, 2020 (the first total lockdown in Italy) and compared them to the same period in 2019. We evaluated the antibiotic prescription rate according to the diagnosis at discharge. The total number of visits significantly decreased (1335 in 2020 vs. 4899 in 2019), as previously reported [7]. Although the rate of antibiotic prescriptions at discharge only slightly decreased (1039 in 2019, 21.2%, vs. 272 in 2020, 20.4%), numerically, antibiotic prescriptions decreased by 73.8%, with antibiotic prescriptions for RTIs accounting for 69% of the total reduction. These data, even though from a single center and not suggestive of a wider antibiotic prescription attitude during the pandemic, may testify to a global reduction in antibiotic prescription in pediatric primary care mainly attributable to reduced visits and reduced RTIs.

Regardless of the underlying motivations, it is possible that, at a larger scale, the reduction in antibiotic prescriptions in pediatrics during the COVID-19 pandemic may have led to a slight reduction in antimicrobial resistance. If confirmed in larger reports, these data suggest that further modifying antibiotic prescription attitudes to reduce prescriptions would likely result in a global reduction in antimicrobial resistance, even over the short term.

In the future, given that it is not going to be possible to reduce the burden of RTIs in children, further education of clinicians on proper antibiotic prescription needs to be implemented and, if possible, guided by point-of-care tests able to discriminate viral from bacterial infections. In this regard, the recent increase in cases of scarlet fever and invasive group A streptococcus (GAS) in the pediatric age group in Europe [8, 9], probably as a consequence of pathogens re-circulating (both GAS and predisposing viruses) after reduced exposure during physical distancing during the COVID-19 pandemic, highlights the need for point-of-care testing to guide treatment of streptococcal infections but at the same time spare unnecessary antibiotics prescription.

## Data Availability

All data generated or analysed during this study are included in this published article.

## Abbreviations

CDC: Centers for Disease Control and Prevention
RTIs: Respiratory tract infections
